# Colored Spectacles

**Published:** 1890-02

**Authors:** 


Colored Spectacles.—Dr. Konigstein, while giving directions in his class on the uses and prescribing of spectacles, said that green glass as a protection against strong rays was worse than useless, and did more harm to a sensitive eye than good, as they allowed the yellow rays to be transmitted and unnecessarily irritate the eye. As a protection against strong rays the blue or smoked glasses were the only real protection. The blue should be light, as a deep blue color produces a clear violet disc in the center of the lens which apparently corresponds to the fovea centralis, and by a protracted use of dark blue spectacles the patient may become annoyed by the mosaic work of the fundus of the eye appearing before him. The phenomenon seems to be connected with the pigment changes in the macula lutea.—*Medical Press and Circular*, Oct. 2, 1889.

